# The fate of rice crop residues and context-dependent greenhouse gas emissions: Model-based insights from Eastern India

**DOI:** 10.1016/j.jclepro.2023.140240

**Published:** 2024-01-05

**Authors:** Emily Urban Cordeiro, Laura Arenas-Calle, Dominic Woolf, Sonam Sherpa, Shishpal Poonia, Kritee Kritee, Rachana Dubey, Amresh Choudhary, Virender Kumar, Andrew McDonald

**Affiliations:** a School of Integrative Plant Science, Soil and Crop Sciences, Cornell University, Bradfield Hall, Ithaca, NY, USA; b CIMMYT-India, Sabajpura, Khagaul, Patna, 801105, Bihar, India; c Environmental Defense Fund, New Delhi, 110001, India; d ICAR Research Complex for Eastern Region, Patna, Bihar, India; e Sustainable Impact Department, International Rice Research Institute (IRRI), Los Baños, Laguna, Philippines

**Keywords:** Soil carbon, Rice residue burning, Life cycle assessment, Greenhouse gases, Climate change

## Abstract

Crop residue burning is a common practice in many parts of the world that causes air pollution and greenhouse gas (GHG) emissions. Regenerative practices that return residues to the soil offer a ‘no burn’ pathway for addressing air pollution while building soil organic carbon (SOC). Nevertheless, GHG emissions in rice-based agricultural systems are complex and difficult to anticipate, particularly in production contexts with highly variable hydrologic conditions.

Here we predict long-term net GHG fluxes for four rice residue management strategies in the context of rice-wheat cropping systems in Eastern India: burning, soil incorporation, livestock fodder, and biochar. Estimations were based on a combination of Tier 1, 2, and 3 modelling approaches, including 100-year DNDC simulations across three representative soil hydrologic categories (i.e., dry, median, and wet).

Overall, residue burning resulted in total direct GHG fluxes of 2.5, 6.1, and 8.7 Mg CO_2_-e in the dry, median, and wet hydrologic categories, respectively. Relative to emissions from burning (positive values indicate an increase) for the same dry to wet hydrologic categories, soil incorporation resulted in a −0.2, 1.8, or 3.1 Mg CO_2_-e change in emissions whereas use of residues for livestock fodder increased emissions by 2.0, 2.1, or 2.3 Mg CO_2_-e. Biochar reduced emissions relative to burning by 2.9 Mg CO_2_-e in all hydrologic categories. This study showed that the production environment has a controlling effect on methane and, therefore, net GHG balance. For example, wetter sites had 2.8–4.0 times greater CH_4_ emissions, on average, than dry sites when rice residues were returned to the soil. To effectively mitigate burning without undermining climate change mitigation goals, our results suggest that geographically-target approaches should be used in the rice-based systems of Eastern India to incentivize the adoption of regenerative ‘no burn’ residue management practices.

## Highlights

• Efforts to build SOC for climate change mitigation, while simultaneously avoiding burning, may have unintended consequences.
• Tier 1, 2, and 3 modelling approaches were used to compare no-burn rice residue pathways.
• Field hydrology had a dominant effect on methane emissions.
• Soil incorporation of residues increased emissions relative to open burning, except in comparatively dry fields.
• Biochar is estimated to have the lowest GHG emissions of the evaluated ‘no burn’ alternatives.

## 1 Introduction

Sustainable development in regions such as Eastern India that have lagged behind during the productivity advances of the Green Revolution era, must pursue approaches that meet both food production and environmental goals in order to avoid or minimize the ecosystem disservices that have undermined sustainability elsewhere in the region (TCI, 2022). Challenges are already emerging, with recent evidence showing that the practice of rice residue burning is not limited to the intensified ‘breadbasket’ region of Northwestern India, but is also on the rise in Eastern India (Urban Cordeiro et al., 2023). Residue burning, particularly in the previously identified burning hotspots in the Northwest, has been widely studied because of its impacts on public health that are associated with small particulate (i.e., PM_2.5_) air pollution (Liu et al., 2018a; Montes et al., 2022; Mor et al., 2022).

In areas where burning is an emerging or established practice, burgeoning carbon offset markets have been proposed as an opportunity to shift the perception of rice straw from a waste product to a valued resource (Gorain et al., 2021). Many current carbon market protocols for GHG mitigation are driven primarily by strategies for increasing soil organic carbon (SOC) stocks. Recycling crop residues to soils directly through incorporation or indirectly through livestock manures likely offers the most viable near-term pathway for increasing SOC in areas where burning is practiced. Nevertheless, from a public policy and ecosystems services perspective, the aim in climate-change mitigation is to reduce net GHG emissions and not to solely build SOC *per se*. Since emissions pathways in rice-based and crop-livestock systems are complex and contingent on the aggregate of methane and nitrous oxide, outcomes from different practice changes are difficult to predict, especially in the context of diverse smallholder systems like those that are common in Eastern India. Methane emissions are the primary GHG of concern in many rice systems, given that flooded conditions promote an anaerobic environment where methanogenic microorganisms are favored (Krüger et al., 2001). From a GHG perspective, some studies suggest that in wet systems, where nitrous oxide is not a major contributor to GHGs (Kritee et al., 2018), methane emissions in rice-based systems can outweigh the benefits from carbon sequestration achieved through building SOC with crop residue recycling (Liu et al., 2014). While *in situ* management of rice residues improve soil properties from an agronomic perspective (Bünemann et al., 2018; Mehmood et al., 2020; Powlson et al., 2011), the net GHG implications of changes in soil carbon management are context-dependent and must be studied from a systems perspective to avoid unintended consequences (Lugato et al., 2018).

No-burn policies that are both responsive to an evolving crop-livestock system (e.g., decreasing herd sizes and dairy commercialization) and create value around straw (e.g., carbon markets) are most likely to reduce the expansion of burning in Eastern India (Urban Cordeiro et al., 2024 (under review)). Yet, the net GHG implications from different residue allocation strategies needs to be more fully understood, particularly considering the complex and varied hydrological conditions that typify rice production systems in the region. Alternative pathways beyond the farm-level exist, including residue use as livestock fodder and biochar creation and return. In Eastern India, rice residues are most commonly used as livestock fodder. Feeding low-quality (i.e., poor digestibility) rice residues is associated with high rates of enteric fermentation (Sirohi and Michaelowa, 2007), a GHG source that accounts for 91% of total methane emissions from livestock in India (Chhabra et al., 2013). Furthermore, some estimates suggest that GHG emissions from livestock products as a whole surpass those from crop production in India (Vetter et al., 2017). The fate of carbon in the landscape and resulting GHG fluxes has not been previously studied in Eastern India. Given the importance of agri-food systems and rice production to overall GHG emissions in India (Crippa et al., 2021; Sapkota et al., 2019; Vetter et al., 2017) and the urgent public health priority of limiting agricultural burning, quantifying the relative GHG implications of different residue management strategies provides essential information for developing robust mitigation options, carbon markets, policy, and field-level decision making.

This research provides a model-based assessment of four different residue management strategies in Eastern India, including burning, *in situ* incorporation, livestock fodder with manure returns, and biochar. It has two primary objectives: (1) to characterize the influence of field hydrology on GHG fluxes in the context of different rice residue management strategies, and (2) to compare net GHG fluxes at the farm-scale from four residue pathways. We hypothesized that wetter production environments would result in greater overall soil-derived emissions compared to drier environments. This increase in emissions was expected to be primarily driven by methane emissions, and we anticipated that the ranking of the four residue pathways would differ based on the hydrologic characteristics. Specifically, we hypothesized that in drier environments, the biochar and incorporation pathways would yield the lowest net GHG fluxes. In wetter environments, we hypothesized that the biochar and burning pathways would result in the lowest net GHG fluxes.

Direct GHG emissions were calculated through Tier 1, 2, and 3 estimation approaches. Our Tier 3 modelling method for soil-based GHG emissions at the field scale uses daily field water measurements across three rice seasons to establish a range of realistic hydrologic boundary conditions to constrain a process-based model (DNDC). As a complement to the dynamic approach used for soil-related emissions, we utilized IPCC Tier 1 and 2 accounting methods to quantify the direct, non-soil GHG emissions from residue burning, biochar production, and bovine livestock feeding. This approach endeavors to expand our understanding of the GHG implications of different rice residue management practices in Eastern India to support the identification and targeting of solutions that address multiple sustainable development goals.

## 2 Methods

### 2.1 Study area

The study is situated in Bihar State in Eastern India. With a mostly rural population of more than 100 million, Bihar's agriculture is characterized by mixed crop-livestock systems and the prevalence of the rice-wheat annual cropping rotation (Erenstein and Thorpe, 2010). West Champaran District (27.08°N, 84.35°E) in the northwest corner of Bihar, where the majority of the study's data originates, is characterized by sandy loam and clay loam alluvial soils, with approximately 77% of farming households cultivating rice-wheat (Ahmad et al., ). The climate of Bihar is dominated by the southwest monsoon that concentrates 85–90% of state's annual average rainfall of 1130 mm (long-term range 630 to 1740 mm in central Bihar) within a four-month period from June–September (Balwinder-Singh et al., 2019). Water is generally abundant during the monsoon season with recurrent flooding of most agricultural fields, but there are large differences in field water conditions between years and across drainage gradients (see Fig. 2). Nearly all rice fields receive supplemental irrigation, but water limitations have been identified as a principal yield constraint to productivity (Balwinder-Singh et al., 2019; McDonald et al., 2023). Rather than differences in irrigation practices, local to regional-scale drainage patterns and soil factors principally govern field hydrology, which uniquely characterizes Bihar in comparison to other northwestern states of the Indo-Gangetic Plain (Singh and Pandey, 2014). Fields within close spatial proximity often have highly varying hydrologic behaviors, ranging from mostly non-flooded to nearly fully flooded over the entire growing season, with many variations in between.

### 2.2 Rice residue management strategies and methods for GHG estimation

**Fig. 1:**
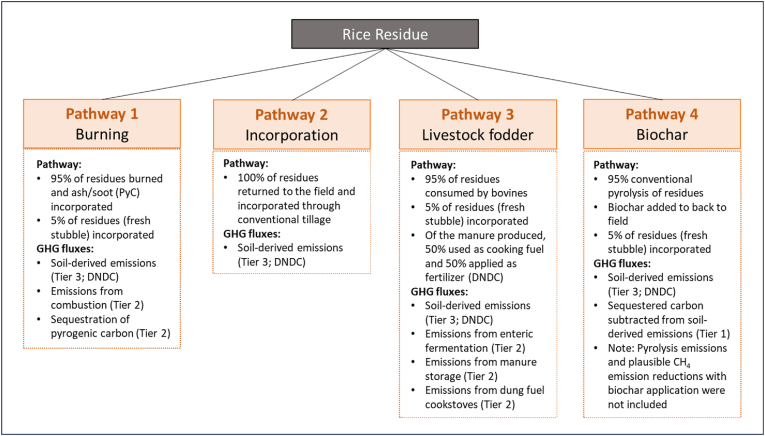
Diagram depicting the four residue fates in this study. All equations are provided in the Methods section of this study.

At the farm scale, four pathways for rice residue management are explored in this study, including burning, *in situ* incorporation, livestock fodder with partial manure return, and biochar (Fig. 1). Details for each of these approaches are provided below. For each pathway, a process-based model (DNDC) was used to quantify soil-based GHG fluxes, with IPCC Tier 1 and 2 accounting methods used to quantify the direct, non-soil GHG emissions of the four residue management strategies. A life cycle assessment (LCA) approach was used, with a limited focus on GHG emissions. By identifying a unit of analysis (i.e., yield from 1 ha of land), drawing boundaries around the process (i.e., farm-level fate of rice residues), and using database inventories to assign values to each impact category (i.e., atmospheric radiative forcing) for each activity in the process, and interpreting the results for its intended audience, an LCA is a valuable ‘decision-support tool’ that translates systems complexity to actionable insights (Horne et al., 2009; ISO, 2006).

**Fig. 2:**
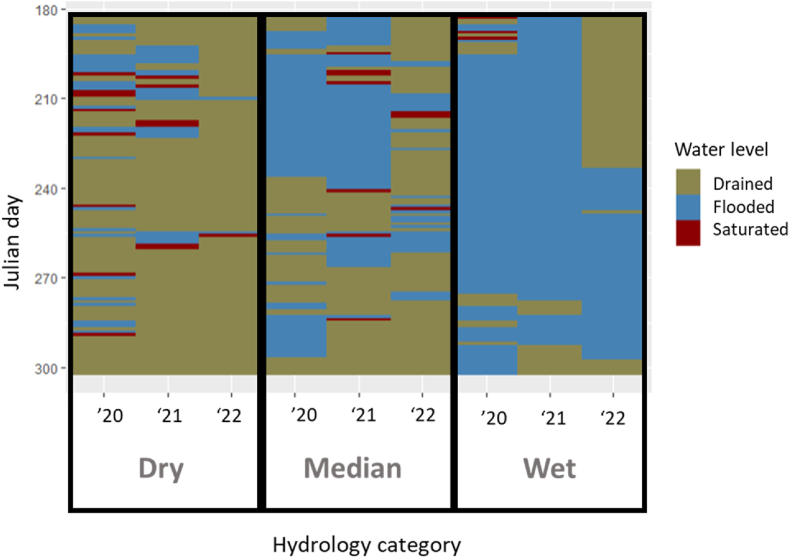
Representative daily field water observations from comparatively dry, median, and wet conditions across three years. These data are used as inputs to DNDC to accurately capture the hydrologic variability that characterizes rice production systems in Eastern India.

### 2.3 The DNDC model: calculating soil-derived GHG emissions with observed hydrologic variability

The DNDC model is a process-based biogeochemical model developed at the University of New Hampshire (University of New Hampshire, 2012). The model is run on a daily time-step using climate, soil, and plant input characteristics to simulate a range of processes including plant growth, soil organic matter dynamics, nitrification, denitrification, and methanogenesis (Li, 2000). We used DNDC v.9.5 to simulate methane (CH_4_) and nitrous oxide (N_2_O) fluxes and soil organic carbon (SOC) over a 100-year period in the rice-wheat rotation for three distinct hydrology categories, namely dry, median, and wet, to provide a range of expected GHG outcomes for each rice residue strategy. Long-term adoption (i.e., 100 years) is required for sequestration permanence, and net GHG balance can shift over time, even from a sink to a source (Lugato et al., 2018). DNDC has been used in other studies to simulate biogeochemical processes and GHG fluxes in rice-based systems (Arenas-Calle et al., 2022; Babu et al., 2006; Zhu et al., 2019) and also to characterize nutrient cycling in the rice-wheat systems of India (Pathak et al., 2006). Wang et al. (2020) reported a correlation (R) between the observed and simulated CH4 of 0.90 (p < 0.01). Pathak et al. (2005) reported a deviation (%) of 3.6 and 6.8 between observed and simulated CH_4_ and N_2_O fluxes, respectively. Arenas-Calle et al. (2022) DNDC model validation results found that simulated and observed net GHG flux had an R^2^ of 0.9. Nitrous oxide was underestimated with an R^2^ of less than 0.1, but only contributed to a small portion of the overall net GHG flux (Arenas-Calle et al., 2022). Babu et al. (2006) validated the DNDC model for emissions in rice and found, across 11 India-based sites, that the percent relative deviation between simulated and observed values ranged from −12.3 to 56.4 for CH_4_ (r^2^ = 0.99) and −247.8 to 28.6 for N_2_O (r^2^ = 0.93). Zhu et al. (2019) stated a correlation coefficient (r) of 0.84 between simulated and observed SOC values with a root mean square Error (RMSE) of 2.75 g kg^−1^.

Typically, biogeochemical models for rice systems rely on fixed assumptions for field water conditions (e.g., ‘flooded’ or ‘alternate wetting and drying’) or, alternatively, use one-dimensional simulation approaches. Neither approach is appropriate for rice production environments like those in Bihar where local to regional drainage patterns and heavy monsoon rainfall exert a strong influence on the field water balance and, in general, supersede the influence of farm management. To account for the range of hydrological differences that characterize rice systems in Bihar, we constrained the DNDC model with observed daily water level data during the rice season (Julian day 183–302) using the ‘water table’ mode in the DNDC model.

Three years (2020, 2021, 2022) of measured rice field water data during the rice growing season were used to create three representative hydrology categories based on the ‘wetness’ distribution of production fields within each year. Year 1 field water observations (2020, average rainy season, *n =* 35) were taken from West Champaran District. Year 2 observations (2021, above average rainy season, *n =* 47) were also taken from West Champaran District. Year 3 observations (2022, below average rainy season, *n* = 183) were taken from nineteen districts distributed across Bihar. Both Year 1 and Year 2 water data were accompanied by comprehensive soil and crop management data.

To collect these data, field water tubes of 10 cm diameter by 30 cm length with perforation holes (5 mm diameter, 2 cm apart) were inserted vertically, 15 cm below the soil surface. Daily water level measurements (−15 to 15 cm) were recorded beginning with rice transplanting and finishing at harvest. Positive values indicate depth of flooding above the soil surface and negative values indicate the depth at which the soil was saturated below the soil surface. Because these data were collected for other research purposes, the three years are not collected from the same field sites. Nevertheless, the range of water regimes characterized within each year (i.e., fully flooded to consistently drained with most fields in-between these extremes) suggests that the data adequately capture the range of field hydrologic possibilities that are representative of the variability within the region as a function of different climate years.

To define a representative range of field hydrology conditions to use as input data for the DNDC simulations, individual production fields from each set of annual field water observations were characterized by the number of flooded days versus non-flooded days. The site with the median value was then selected to represent the ‘median’ hydrology category for that year. The ‘dry’ and ‘wet’ representative sites were randomly selected from all sites one standard deviation away from the mean in both directions, resulting in nine distinct hydrologic regimes (i.e., 3 categories x 3 years) that were used as inputs for DNDC simulations. Thereafter, 100-year DNDC water level input files were constructed for each hydrologic category (i.e., dry, median, wet) by randomly selecting a climate year (i.e., 2020, 2021, 2022 – with equal probabilities of selection) to use as the field hydrology for a given simulation year. For the wheat season in all simulations, water level input files were set to −100 cm and to 0 cm on Julian days 22, 340, and 352 to correspond to typical farmer irrigation practices. The potential influence of climate change and irrigation practice changes on field hydrology are unknown, hence were not considered in our simulations.

Data inputs required for DNDC included climate, soil, management, and crop data. Given that only 2020 and 2021 hydrology data included crop management and soil data, these associated parameters from the six hydrologic sites (three from 2020 and three from 2021) were averaged to create a ‘synthetic site’ for the DNDC runs. Daily climate data were obtained from the NASA Langley Research Center (LaRC) POWER Project, and included maximum and minimum temperature (°C), wind speed at 2 m (m s^−1^), and humidity (%). Daily temperature data corresponding to each representative field (i.e., for each hydrologic category x year) were used as a model input. Precipitation was set to zero during the rice season, as water availability was controlled by the water level files.

The DNDC crop model was calibrated through adjustment of the following parameters: maximum biomass (kg C ha^−1^) (grain, leaves and stems, and roots), biomass fraction (fraction of grain, leaves and stems, and roots), biomass C:N ratio (grain, leaves and stems, and roots), total N demand (kg N ha^−1^), thermal degree days (TDD °C) (cumulative temperature from seeding to maturity), water demand (g water per g dry matter), and N fixation index (default value of 1 for non-legume crops) (Partridge et al., 2011). Initially, wheat optimum temperature, wheat biomass fractions and C:N ratios of grain, leaves and stems, and roots were taken from Wang et al. (2023). For rice, biomass fractions were taken from Kar et al. (2021) and optimum temperature from Dhanushka (2011). The crop model parameters where then manually calibrated following the protocol of Partridge et al. (2011).

The manual calibration process began with adjustment of the maximum biomass parameter by adjusting the grain biomass C according to observed maximum grain yields. The maximum observed rice and wheat yields of the most common varieties were taken from 2021 crop management data (*n* = 210, West Champaran District). The maximum observed value for the most common rice variety, Sarju52, was 5 Mg ha^−1^ (mean: 3.77 Mg ha^−1^). The maximum observed value for the most common wheat variety, UP262, was 4.87 Mg ha^−1^ (mean: 3.39 Mg ha^−1^). The biomass C parameters of leaves, stems, and roots were based on grain biomass C and associated biomass fractions. Final biomass C parameters used in the study are available in Table S1. To begin the calibration process, non-limiting irrigation and N fertilization rates were provided. Simulated and observed yields were compared in year 3 of the simulations. From preliminary simulations, yield levels stabilized around year 3. Rather than conducting a spin-up process that would require a series of state variables that were not available for Eastern India, we used the initial year (i.e., year 3) where yields reached a stable state.

The grain biomass C parameter was increased incrementally until the simulated yields and observed maximum grain yields were within 10% of one another. Next, the seasonal accumulative thermal degree days (TDD) were incrementally adjusted from the starting value so that the simulated crop maturity date matched the harvest date. For example, initially, the crop maturity date was earlier than the harvest date, so the TDD value was incrementally increased. This approach ensured that the crop model parameters enabled the highest observed yields for the region from our data. The long-term DNDC simulations were conducted assuming that average ‘current’ yield levels were maintained, hence fertilizer was reduced until the output grain biomass C was within 10% of the rice and wheat observed mean grain yields.

Measured field data were used for initial soil parameters: SOC (0–10 cm): 5.2 g C kg^−1^ soil; pH: 8.12; bulk density: 1.46 Mg m^−3^; soil texture: sandy clay; and the clay fraction: 0.42 (see Table S1). Crop management parameters were also based on measured field data: planting dates: June 9 (rice nursery) and November 23 (wheat); harvesting dates: October 29 (rice) and April 10 (wheat); and tillage (two passes before crop establishment with 20 cm and 10 cm soil disturbance for rice and wheat, respectively).

### 2.4 Calculating non-soil derived, direct GHG emissions

Direct GHG emissions that were non-soil derived were estimated via a series of Tier 1 and Tier 2 approaches. For these estimates, the biomass of rice straw residue was estimated using Eq. (1).

(1) $$Y_{\text{rs}}=Y\;*\;R$$

Where.

Y_rs_ = rice straw (t ha^−1^, fresh)
Y = rice grain yield (t ha^−1^)
R = crop production to straw ratio

For all rice residue management strategies, the amount of straw produced was estimated using current rice yields (3.77 t ha^−1^) and held constant throughout all simulation years. R was taken as 1.5 (Jain et al., 2014).

#### 2.4.1 Livestock fodder pathway: feeding rice residues

Direct GHG emissions from the livestock pathway include GHG fluxes in soils through manure application (as modeled in DNDC), enteric emissions (primarily belching), burning manure as a cooking fuel source, and manure storage, as these sources represent the majority of emissions (Garg et al., 2016). Our findings through qualitative interviews in Buxar district, Bihar state for a previous study (Urban Cordeiro et al., 2024 (under review)) found that approximately half of the bovine manure produced at the household level is placed on the field and approximately half is dried and used for cooking fuel.

The amount of enteric methane produced by a representative herd model for IGP based on consumption of all the rice straw yield was calculated using Eq. (2).

(2) $$E_{\text{CH}4}=Y_{m}\;*\;Y_{\text{rs}}\;*\;E_{s}\;/\;E_{\text{CH}4}$$

Where.

E_CH4_ = CH_4_ emissions (kg CH_4_ ha^−1^ yr^−1^)
Y_m_ = CH_4_ conversion factor based on herd model (dimensionless)
Y_rs_ = rice straw yield (kg DM ha^−1^ yr^−1^)
E_s_ = energy content of rice straw (HHV MJ kg^−1^ DM)
E_CH4_ = energy content of methane (MJ kg^−1^)

A representative herd model for IGP was comprised from the 20th Livestock Census (Livestock census. 20th, 2019), in which the average percent of each livestock category (i.e., age, sex, type, milking status) was estimated. Because each livestock category has a different methane conversion factor (Swamy and Bhattacharya, 2006), each factor was weighted according the representative herd model composition to arrive at the final derived conversion factor (Y_m_) of 4.74, as explained as a Tier 2 methodology of IPCC. E_s_ was taken as 14.08 MJ kg^−1^ (Gummert et al., 2019) and E_CH4_ was taken as 55.65 MJ kg^−1^ (IPCC et al., 2006).

The amount of manure produced, assuming all rice straw on a per area yield basis was consumed, by bovines was calculated using Eq. (3) (modified on a per area yield basis, IPCC et al., 2006).

(3) $$VS=\left[*\left(1-DE\right)+\left(UE*\right)\right]*\left[\left(\frac{1-ASH}{18.45}\right)\right]$$

Where.

VS = volatile solid excretion on a dry-organic matter basis (kg ha^−1^ yr^−1^)
GE = gross energy of straw consumed (MJ ha^−1^ yr^−1^)
DE = digestible energy fraction
(UE * GE) = urinary energy expressed as fraction of GE
ASH = ash content of manure expressed as a fraction of the dry matter feed intake

The digestible energy fraction (DE) was taken as 0.55 as the given coefficient for the Indian Subcontinent (IPCC et al., 2006), with UE = 0.04 for most ruminants and ASH = 0.08 for cattle from IPCC et al. (2006). Gross energy of straw consumed was calculated using Eq. (4).

(4) $$\text{GE}=Y_{\text{rs}}\;*\;E_{s}$$

Where.

GE = gross energy of straw consumed (MJ ha^−1^ yr^−1^)
Y_rs_ = rice straw yield (kg ha^−1^ yr^−1^)
E_s_ = energy content of rice straw (MJ kg^−1^)

The energy content (HHV) of rice straw (E_s_) was taken as 14.08 MJ kg^−1^ DM (Gummert et al., 2019). Of the manure produced (volatile solids), it was assumed that half was placed back on the field five days before the start of the rice season and half was burned in cookstoves. For field application in DNDC, the percent nitrogen in the manure was taken as 2.2% (dry and ash-free basis), a conservative value was selected given the low intensity livestock systems of this region (Font-Palma, 2019).

Tier 2 emission estimates for CH_4_ and N_2_O were applied to manure storage (Garg et al., 2016), as farmers store manure daily until it is taken out to the field before dry tillage pre-monsoon or used as cook fuel. CH_4_ and N_2_O emissions were calculated with Eq. (5) and Eq. (6), respectively (IPCC et al., 2006).

(5) $$E_{\text{CH}4‐\text{manure}}=\text{VS}\;*\;B_{o}\;*\;0.67\;*\;\text{MCF}$$

Where.

E_CH4-manure_ = CH_4_ emissions from manure storage on a per area yield basis (kg)
VS = total volatile solids excreted on a per area yield basis (kg dry matter)
B_o_ = maximum methane producing capacity of manure excreted (m^3^ CH_4_ kg^−1^ of VS excreted)
MCF = methane conversion factor for solid manure storage system

B_o_ was taken as 0.13 m kg^−1^ VS for dairy cattle on Indian Subcontinent (IPCC et al., 2006). The conversion factor of 0.67 kg m^−3^ is used to convert m^3^ CH_4_ to kg CH_4_. MCF was taken as 0.05 for ‘warm’ annual average ambient temperature (Garg et al., 2016).

(6) $$E_{N2O‐\text{manure}}=N_{f}\;*\;\text{VS}\;*\;{\text{EF}}_{n}\;*\;44/28$$

Where.

E_N2O-manure_ = direct N_2_O emissions from manure management system (kg N_2_O)
N_f_ = fraction of N in volatile solids excreted (dry and ash-free basis)
VS = total volatile solids excreted on a per area yield basis (kg dry matter)
EF_n_ = emission factor for direct N_2_O emissions from manure management system (kg N_2_O–N/kg N)

N_f_ was taken as 0.022 (dry and ash-free basis) (Font-Palma, 2019). EF_n_ was taken from Ippolito et al. (2020). The conversion of 44/28 converts N_2_O–N emissions to N_2_O emissions.

Tier 1 emission estimates for CH_4_ and N_2_O were applied to dung fueled cookstoves in Eq. (7) (IPCC et al., 2006).

(7) E=FC*EF

Where.

E = emission of a given GHG by type of fuel (kg GHG)
FC = amount of fuel combusted (TJ)
EF = emission factor (kg gas TJ^−1^)

The emission factors taken for CH_4_ and N_2_O were 281 kg TJ^−1^ and 27 kg TJ^−1^, respectively (IPCC et al., 2006). The amount of fuel combusted for manure (FC) was found using Eq. (8).

(8) FC=NCV*M

Where.

FC = The amount of fuel combusted (TJ)
NCV = The net caloric value (NCV) of cattle dung (TJ kg^−1^)
M = mass of cattle dung on dry-organic matter basis (kg)

The net caloric value (NCV) of cattle dung was taken as 17.8 MJ kg-1 (converted to TJ kg^−1^) for dry manure (Thygesen and Johnsen, 2012).

#### 2.4.2 Biochar pathway: pyrolysis of rice residues

The net avoided GHG emissions (CO_2_-e) were summarized using Eq. (9) (Woolf et al., 2021). Biochar was not included within the DNDC simulations, but instead the net avoided GHG emissions were subtracted *ex post facto*. These estimates excluded the CH_4_ abatement effect of biochar additions. Due to the lack of long-term studies, it is unknown if this effect persists beyond a few seasons and, therefore, was excluded.

Biomass pyrolysis can be conducted using a wide range of technologies. One difference between processes involves how the heat is supplied to the biomass feedstock. The most common heating method is indirect heat through the walls of an enclosed vessel from which air is excluded. Other means of heating include, for example, microwave pyrolysis, or direct heating by using high temperature particulate solids (such as sand) or fluids (which may be either gases, e.g., in a fluidized bed, or liquids, e.g., in a molten salt reactor) (Boateng et al., 2015). The heating rate is also an important factor, with processes generally classed as either fast pyrolysis (in which finely divided biomass is rapidly heated), or slow pyrolysis which can accommodate larger particle sizes and lower heat-transfer coefficients. Fast pyrolysis is typically employed in the production of liquid biofuels, with biochar being a by-product and generally has higher capital costs than slow pyrolysis (Tan et al., 2021). Other than heating rate, the other critical parameter in determining biochar properties and yields is the pyrolysis temperature (Ippolito et al., 2020). We assumed that pyrolysis will be conducted at a “medium temperature” using the temperature-range classifications adopted in Woolf et al. (2021), in which medium temperature is defined as pyrolysis between 450 and 600 °C. This temperature range is typical of most current pyrolysis equipment. Temperatures above 600 °C typically require higher cost materials to withstand the conditions, greatly increasing the capital cost (Woolf et al., 2017). Temperatures below 450 °C, on the other hand, generate a less persistent biochar product with a greater labile carbon fraction that can have adverse impacts on crop production such as leading to nitrogen immobilization. Temperatures lower than 350 °C (such as in torrefaction and hydrothermal carbonization) do not produce a residue that is sufficiently carbonized or recalcitrant to be considered as biochar (Deenik et al., 2010; Liu et al., 2018b).

(9) $${\text{GHG}}_{\text{bc}}=M_{\text{bc}}\;*\;F_{\text{perm}}\;*\;44/12$$

Where.

GHG_bc_ = net avoided GHG emissions in units of CO_2_-equivalent (CO_2_-e)
M_bc_ = mass of biochar C added to soil (kg)
F_perm_ = the fraction of biochar organic carbon remaining after 100 years
44/12 = conversion factor from C to CO_2_-e

The original published equation (Woolf et al., 2021) also includes avoidance of N_2_O emissions, but because our biochar application rate is below 10 Mg C ha^−1^, N_2_O was negligible in this context. Because the amount of organic carbon in the biochar and the pyrolysis temperature may vary, the estimate of F_perm_ is 0.71 (SE: 0.03), where we assume medium-temperature pyrolysis is used and the modeled permanence of the biochar is evaluated over 100 years at the mean annual temperatures of global croplands (14.9 °C) (Woolf et al., 2021). M_bc_ was found by multiplying the biochar yield faction (F_by_) by the rice straw yield (Y_rs_) as calculated in Eq. (10).

(10) $$M_{\text{bc}}=F_{\text{by}}\;*\;Y_{\text{rs}}\;*\;D$$

Where.

M_bc_ = mass of biochar C added to soil (kg)
F_by_ = biochar C yield faction (dry, ash-free)
Y_rs_ = yield rice straw (kg)
D = dry matter fraction of fresh rice straw

D was taken as 0.86 (Jain et al., 2014). Biochar C yield faction (dry, ash-free) (F_by_) was found through an empirical regression model as a function of pyrolysis temperature T (K) and feedstock lignin mass fraction (L_f_) in Eq. (11) (Woolf et al., 2014).

(11) $$F_{\text{by}}=0.126+0.273\;*\;L_{f}+0.539*\exp\left(‐0.004*T\right)$$

L_f_ was taken as 0.179 for rice residues, given on a dry matter basis (Woolf et al., 2021), as originally provided in the Phyllis2 database (ECN, 2021). T assumes a medium pyrolysis temperature of 798.15 K (525 °C), as pyrolysis between 450 and 600 °C is considered a medium temperature range (Woolf et al., 2021).

#### 2.4.3 Burning: *in situ* open burning of rice residues

The total GHG emissions N_2_O and CH_4_ from burning were found using Eq. (12) (IPCC et al., 2006).

(12) $$E=R_{\text{mb}}\;*\;\text{EF}$$

Where.

E = total emissions of the species (kg)
R_mb_ = mass of rice residue burned (kg)
EF = emission factor (g kg^−1^)

EF (g kg^−1^) of rice residue burning for N_2_O was taken as 0.48 (Sahai et al., 2007) and EF for CH_4_ was taken as 9.59 (Ravindra, Singh and Mor, 2019a). Both are Tier 2, country-specific emission factors. The mass of rice residue burned (R_mb_) found using Eq. (13), applied by (Ravindra, Singh and Mor, 2019b).

(13) $$R_{\text{mb}}=Y\;*\;R\;*\;D\;*\;B$$

Where.

Y = rice yield (kg ha^−1^)
R = production to residue ratio
D = dry matter fraction
B = burn efficiency fraction

R was taken as 1.5 (Jain et al., 2014). We assumed all available crop residue was burned. D was taken as 0.86 (Jain et al., 2014). The burn efficiency fraction (B) was taken as 0.89 (Turn et al., 1997). Thus, we can assume 11% of carbon remains in pyrogenic carbon (PyC) form. PyC encompasses a continuum of pyrogenic organic material (charred biomass, charcoal, and soot), and while mean residence time of these carbon forms vary in the environment, most PyC is relatively stable across decadal or centennial timeframes (Bird et al., 2015; Santín et al., 2016). As such, the net avoided GHG emissions (CO_2_-e) through PyC were summarized using the previous biochar equation (Eq. 9). The mass of biochar C added to soil from burning (M_bc_) was found using Eq. (8) and multiplying the fraction of pyrogenic carbon remaining after burn (P_f_) and the fraction of remaining PyC *in situ* (P_r_) (i.e., excluding atmospheric PyC (black carbon) transported in smoke) (Eq. (14)).

(14) $$M_{\text{bc}}=F_{\text{by}}\;*\;Y_{\text{rs}}\;*\;D\;*\;P_{f}\;*\;P_{r}$$

D is taken as 0.86 (Jain et al., 2014). P_f_ is the inverse of the burn efficiency fraction (B = 0.89; Turn et al., 2017) of 0.11. P_r_ is the fraction of PyC remaining on site and taken as 0.9895 (Santín et al., 2016). There is a large uncertainty around the percent of black carbon released into the atmosphere, so this calculation only accounts for estimates of PyC that remain on site.

#### 2.4.4 Conversion of GHG fluxes to a common unit

The total CH_4_ and N_2_O emissions were converted to their CO_2_-equivalent (CO_2_-e) using Eq. (15).

(15) $$T_{\text{CO}2e100}=\text{CH}4\;*\;{\text{GWP}}_{N2O}+N2O\;*\;{\text{GWP}}_{\text{CH}4}$$

The global warming potentials of these non-CO_2_ gases were taken as 27.9 and 273 for CH_4_ and N_2_O for a 100-year period (IPCC, 2021).

## 3 Results

### 3.1 Characterizing the range of rice hydrologic conditions in Eastern India

The daily measured water conditions from the nine representative sites used for each hydrologic category (i.e., dry, median, wet) in the DNDC model simulations are presented in Fig. 2. Drained conditions, depicted in brown, denote days when a free water surface is below 15 cm depth. Saturated conditions, depicted in red, denote days without flooding but with saturated conditions within the top 15 cm of soil. Flooded conditions, denoted in blue, represent days with ponded water above the soil surface. The range of observed conditions is extreme, with ‘wet’ fields in a high rainfall year like 2021 almost always flooded and ‘dry’ fields in a drier year like 2022 almost continuously drained. The most representative ‘median’ sites are characterized by a higher number of transitions between flooded and drained conditions, but this too depends on the nature of the climate year and the interaction between precipitation patterns and landscape drainage factors. Note that the representative sites for 2020 and 2021 are from a single district, reflecting the high level of short-range variation in field water conditions. In 2020 and 2021, most fields were irrigated during land preparation, before daily field water measurements began. Most farmers irrigated during the 2022 growing season, as it was a very dry climate year and data was derived from across Bihar state with varying site characteristics. Given the central influence of the soil water environment on greenhouse gas emissions in rice (Nikolaisen et al., 2023), our results suggest that accurate GHG estimations in Eastern India cannot be developed without accounting for hydrologic complexity.

### 3.2 Field hydrology, *in situ* recycling of crop residues, and methane emissions

**Fig. 3:**
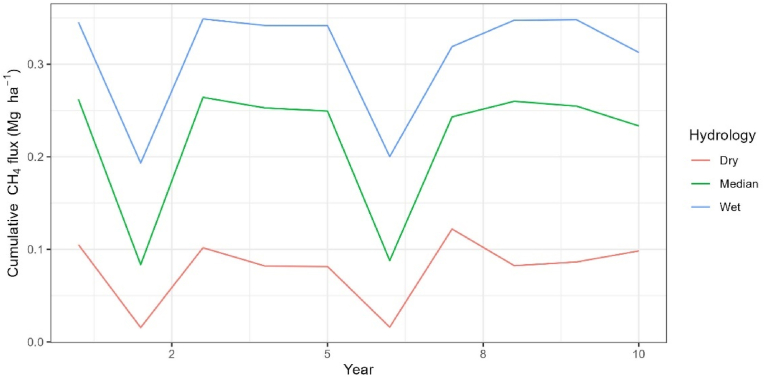
DNDC simulated emissions from rice systems across three hydrology categories. Simulations are conducted for a 10-year period with no recycling of crop residues.

Results of shorter-term (10-year) DNDC methane emissions simulations are presented to visualize patterns of interannual variability of fluxes based solely on contrasting hydrologic conditions with no crop residue returns (Fig. 3). Mean annual CH_4_ emission were 0.08, 0.22, and 0.31 Mg ha^−1^ over the 10-year period, with the wetter production environment consistently producing approximately 3-times more CH_4_ than the dry environment with emissions from the median production environment more closely resembling the wet sites in high rainfall years (e.g., simulation year 3) and the dry sites in low rainfall year (e.g., simulation year 6). Variability between years was also high with CH_4_ emissions in the median hydrology scenario more than doubling in a wetter climate year compared to a drier climate year. To generate robust baseline GHG emission estimates for Eastern India with measured field data, model results highlight the need for multi-year assessments taken across hydrologic gradients.

**Fig. 4:**
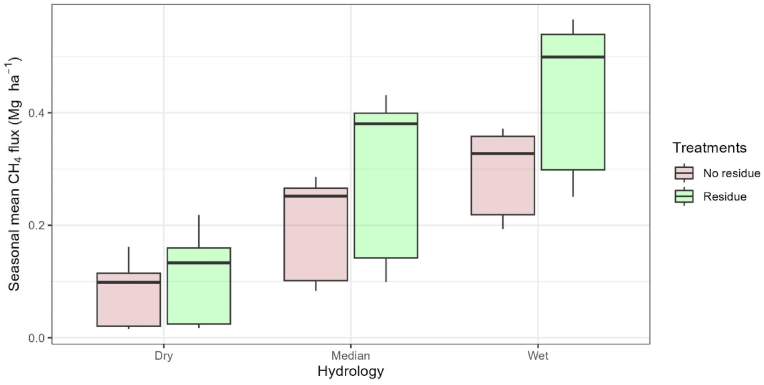
Annual rice season methane flux from 100-year DNDC simulations. Simulations contrast emissions for the three hydrologic classes with and without *in situ* residue recycling.

Given the well-established linkages between soil carbon and methane production, we then explored the interactions between rice field hydrology, rice residue recycling, and the methane emissions over a 100-year simulation (Fig. 4). For all hydrologic categories, recycling of crop residues increased methane emissions, but these increases were much lower in relative and absolute terms in the dry rice production environment. In the treatments with no residue return, the mean seasonal CH_4_ fluxes from the median and wet categories were 2.5 and 3.6 times higher than those from the dry category. With full residue return, the mean seasonal CH_4_ fluxes from the median and wet categories were 2.8 and 4.0 times higher than those from the dry category.

Within each hydrologic class, the influence of residue on CH_4_ fluxes was more variable from year-to-year in the median and wet production environments. In the dry environment, the CH_4_ implications for residue return were small (mean of 0.08 Mg ha^−1^ without residue; mean of 0.11 Mg ha^−1^ with residue) with a 37.5% (0.03 Mg ha^−1^) mean annual methane flux difference between no residue (NR) and residue return. This difference increased to 48.1% (0.10 Mg ha^−1^) and 48.0% (0.14 Mg ha^−1^) in the median and wet hydrology categories, respectively.

### 3.3 Rice residue management strategies and farm-scale GHG emissions

Net GHG emissions associated with N_2_O, CH_4_, and soil carbon sequestration were estimated for the four rice residue management strategies across three hydrologic production environments for rice through a combination of Tier 1, 2, and 3 modelling approaches. Soil-based net emissions were added to non-soil emissions to estimate total direct GHG fluxes (Table 1) and are presented as annual mean values over a 100-year period. With the exception of biochar, differences across the hydrologic categories were substantially larger than net emission differences among the rice residue management strategies within each hydrologic class. Residue burning was estimated to result in lower net emissions than *in situ* residue recycling in all but the dry environment. Net emissions from residue recycling were on par with the livestock strategy in the median and wet environments. However, in the dry rice environment, the livestock strategy was estimated to generate around twice the emissions of either burning or recycling. Modest net emission advantages were characterized by the livestock strategy (11.0 Mg CO_2_-e) compared to recycling (11.8 Mg CO_2_-e) in the wet environment.

**Table 1:** Net greenhouse gas emissions for four rice residue management strategies.

	Pathway	Season average GHG fluxes from soils (Mg CO_2_-e)			Other direct non-soil GHG fluxes (Mg CO_2_-e)	Total direct GHG fluxes (Mg CO_2_-e)		
		Dry	Median	Wet		Dry	Median	Wet
1	Burning (*in situ*)	2.1	5.7	8.3	0.4	2.5	6.1	8.7
2	*In situ* incorporation	2.3	7.9	11.8	0	2.3	7.9	11.8
3	Livestock fodder + manure return	2.2	6.0	8.8	2.2	4.5	8.2	11.0
4	Biochar	2.1	5.7	8.3	−2.5	−0.4	3.2	5.8

For all residue management pathways, total GHG emissions increased toward a more net positive flux as environments became wetter. Soil CH_4_ had the largest flux for all pathways and all hydrology categories, except for the biochar pathway in the dry hydrology category. Negligible fluxes were grouped as ‘other’, which included soil N_2_O in all pathways, manure and cookstove emissions from the livestock pathway, and combustion emissions from the burning pathway (Table S4).

**Fig. 5:**
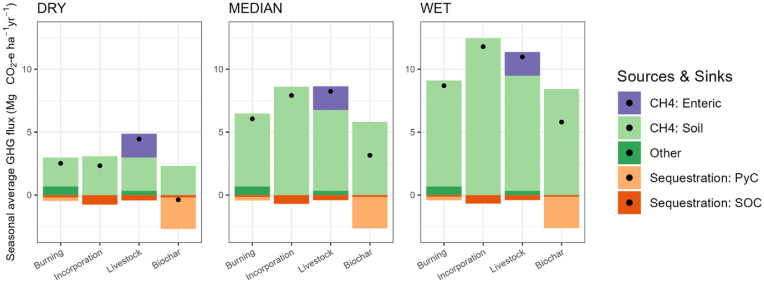
Negligible fluxes were grouped as ‘Other’, which included soil N_2_O in all pathways, manure and cookstove emissions from the livestock pathway, and combustion emissions from the burning pathway. *Values for ‘Other’ are provided in*Table S4.

Both residue incorporation and livestock fodder pathways had higher net GHG emissions than residue burning in all cases, except for incorporation in dry environments which was comparable to (slightly lower than) burning (Fig. 5). In all environments, the biochar pathway had the lowest net GHG emissions, although these were only net negative in the dry sites. In all other scenarios, CH_4_ emissions outweighed the SOC sequestration potential.

In dry environments, the livestock pathway had the highest GHG emissions but the difference between livestock fodder and residue incorporation strategies became negligible as the environments became wetter. In wet environments, the negative GHG implications of the livestock pathway are slightly less than *in situ* incorporation despite other prospects for GHG creation associated with livestock, including enteric fermentation, manure storage, and dung-fueled cookstoves.

### 3.4 DNDC model comparisons

In comparison with prior field studies in rice systems, our simulated time-averaged emissions of 83–447 kg CH_4_ ha^−1^ yr^−1^ and 0.010–0.020 kg N_2_O ha^−1^ yr^−1^ (equivalent to 62–335 kg C ha^−1^ yr^−1^ and 0.007–0.013 kg N ha^−1^ yr^−1^, respectively) generally fall within the measured emission ranges from field studies in the South Asia. The higher end of these ranges corresponds to simulations with residue return, which show slightly higher CH_4_ emissions than typical of published field studies which often do not include full residue return. A field study across 11 rice-based systems in India measured annual CH_4_ emissions ranging from 4.6 to 436.5 kg C ha^−1^, and also found that DNDC adequately simulates seasonal fluxes (Babu et al., 2006). Other studies from South India have recorded lower CH_4_ emissions, with monsoon season fluxes ranging from 113.5 to 164.5 kg CH_4_ ha^−1^ and 0.73–1.23 kg N_2_O ha^−1^ (Oo et al., 2018). Another study argued that N_2_O fluxes in intermittently-flooded rice systems have been largely underestimated and not given enough attention in GHG inventories, having found measured seasonal N_2_O fluxes ranging from 0 to 33 kg N_2_O ha^−1^ in their India-based field study (Kritee et al., 2018). DNDC does not simulate N_2_O in flooded conditions (Babu et al., 2006), but since many of our simulations did not have persistent flooding, the reasoning is not clear. Given the inevitable uncertainties with both modeled and measured GHG emission estimates, our results are best understood as indicative values that suggest the relative emission differences that are generating by rice residue practices and hydrologic variation.

DNDC uncertainty and sensitivity analyses are included in the Supplementary Information.

## 4 Discussion

Agricultural burning in South Asia is a major concern for public health from PM_2.5_ emissions (Liu et al., 2018a; Montes et al., 2022; Mor et al., 2022). While it is useful to compare this practice to other rice residue management strategies from the perspective of GHG emissions, avoiding the burning of crop residues must remain a policy imperative independent of implications for climate change mitigation. Beyond the boundaries of the current study, future research should emphasize comprehensive life cycle assessments that compare positive and negative consequences for different rice residue management strategies, including air quality and agronomic benefits derived from *in situ* residue recycling in diverse crop production contexts.

The variation in GHG emissions among the four strategies—burning, soil incorporation, livestock fodder, and biochar—primarily resulted from soil-derived methane emissions. Wetter sites resulted in a general rise in methane emissions across all scenarios due to the proliferation of anoxic conditions in the soil, facilitating methane production through methanogenesis. The introduction of organic amendments, such as the incorporation of residues into the soil or the partial return of livestock manures, notably augmented soil-derived methane emissions within these scenarios. Various studies (Tang et al., 2014; Zou et al., 2004) have demonstrated that the retention of post-harvest residue intensifies methane emissions. The interaction between crop residues and the hydrological context will determine if the decomposition of organic compounds is carried out in aerobic (well-aerated soils) or anaerobic (flooded soils) conditions, thus influencing methane production. In the context of the livestock pathway, methane emissions originating from enteric fermentation constituted the second largest source. This emission source, substantial in livestock systems, is influenced by diet and herd type. For instance, in India, enteric fermentation contributes to 91% of total methane emissions from livestock (Chhabra et al., 2013), primarily attributed to the feeding of low-quality rice residues with poor digestibility (Sirohi and Michaelowa, 2007).

Despite the fact that carbon offset markets have previously been suggested as an opportunity to incentivize more sustainable crop production practices (Gorain et al., 2021), our results suggest that the most commonly supported strategy of building SOC stocks will typically increase rather than decrease emissions in the context of rice-based agricultural systems (with the possible exception of residue incorporation in dry sites). In Eastern India, some fields remain persistently flooded due to factors like landscape-scale drainage patterns that are not readily controlled by farmers. In our study when rice residues are recycled, these (‘wet’) fields are predicted to generate almost 50% more GHG emissions compared to average (‘median’) hydrologic production environments, an observation that is consistent with measured field data (Liu et al., 2014; Xu et al., 2015). Over a 100-year timeframe, the countervailing GHG emission effects of increasing SOC stocks with residue recycling were estimated to be very small in our simulations since stock increases diminish significantly with time (Lugato et al., 2018). Other research from India suggests that the use of conservation agriculture for *in situ* management of rice residues (i.e., retention at the soil surface) can reduce CH_4_ emissions when residues are recycled (Sapkota et al., 2015), but it is important to note that conventional tillage with residue incorporation is the most feasible near and medium-term option in Eastern India given the slow adoption rates of conservation agriculture compared to elsewhere in the South Asia region where investments have been strong and farmers are better positioned to make capital investments in new equipment (Keil, D'souza, & McDonald, 2015).

Our full accounting of emissions for crop residue cycling suggests that carbon offset markets in Eastern India must look beyond their current emphasis on increasing soil carbon storage to reduce GHG emissions in rice-based systems. As such, the opportunity to use carbon financing as a mechanism to disincentivize burning appears to be limited in Eastern India. Biochar, however, does appear to offer significant promise as a mitigation strategy across hydrologic production environments. Some field studies also suggest that biochar may also reduce CH_4_ emissions in soils (Jeffery et al., 2016), by as much as 72% in upland rice and 12% in paddy rice (Ji et al., 2018); these processes are not currently simulated in DNDC, hence we may be underestimating the mitigation value of biochar. Although the technical potential of biochar is compelling, economic feasibility, social desirability, and off-farm emission costs of biochar are not accounted for in this study. For example, the cost of biochar in its ready-to-use form significantly hinders its accessibility for low-income farmers. We also assumed “clean” pyrolysis equipment with negligible emissions and negligible emissions from biomass transport. Future feasibility studies and extension strategies for biochar in Eastern India should consider the full costs, economic and environmental, of small-scale and large-scale infrastructure options (Bhatnagar et al., 2022) together with an understanding of labor and other potential constraints for biochar production at the household and community levels (Müller et al., 2019).

Aside from biochar, our results do not suggest that residue recycling or livestock feeding as viable strategies for reducing net GHG emissions compared to burning. Nevertheless, measured differences in field hydrology were found to have a profound impact on methane emissions and, consequently, net emissions, a result consistent with established principles (Krüger et al., 2001). Our results suggest that rice residue used as fodder from comparatively dry fields results in higher net GHG emissions than alternative strategies, suggesting that geographic targeting criteria can also be used to set priorities for livestock production. While recurrent drainage (i.e., ‘alternate wetting and drying’ – AWD) is often a suggested approach to decreasing CH_4_ emissions in rice (Liu et al., 2014), farmers often lack the ability to control water during the monsoon season when inundating rainfall and landscape-scale controls on drainage are the principle governing factors of the field water environment (Bonsor et al., 2017). In Eastern India, mitigation strategies for rice-based systems should likely shift away from water management and SOC increase to landscape characterization that identifies comparatively ‘safe’ places to retain carbon in the landscape to limit CH_4_ emissions, with *ex situ* residue management options prioritized for ‘risky’ environments that are intrinsically prone to generate methane. On-going research by our group in Eastern India seeks to characterize and predict the long-term hydrologic behavior of rice fields in the region to help guide such efforts. Similar studies are needed across the principal rice producing regions of South Asia where monsoon season rice crops are cultivated.

Beyond farm-scale analysis of GHG emissions for rice residue management alternatives, comprehensive LCAs are needed that include other impact categories, such as air quality, food security, and livelihood considerations. Ideally, these would be ‘consequential’ LCAs that also account for indirect impacts on factors such as land use changes (Schmidt, 2008), and would aggregate evidence for multiple impact categories while expanding the system boundary to include activities such as tractor fuel used for residue incorporation, strategies for moderating enteric fermentation, transportation costs related to biochar production, and black carbon emissions from burning. Black carbon emissions have a substantial impact on increasing the amount of solar radiation absorbed by the glaciers in the Himalayan region, thus raising temperatures and increasing glacier melt. Nearly 3% of the atmospheric black carbon impacting the Himalayan region is derived from agricultural burning in South Asia (Alvarado et al., 2018).

This study has multiple limitations and recommendations. Climate change and plausible future changes in irrigation practices were not considered in this study and should be explored in future studies. There are pathways for rice residue management that exist beyond the farm-scale that were not included in this study, such as bioethanol, mushroom production, and uses in the paper industry (Dutta et al., 2022). We recommend that additional pathways are included in future studies. A diverse set of ecosystem services were not quantified in this study, including the potential co-benefits of building SOC in soils from an agronomic perspective (Bünemann et al., 2018; Powlson et al., 2011). Although there is evidence that our DNDC modelling approach may underestimate N_2_O emissions (Arenas Calle et al., 2022; Kritee et al., 2018), since the overall contribution of N_2_O to net GHGs was negligible, improved estimates of this flux are unlikely to alter the main conclusions substantively. In the livestock pathway, there are multiple potential impacts that are very difficult to quantify. As liquefied petroleum gas (LPG) cookstoves are promoted in rural India, the ratio of manure used for cooking versus soil improvement is likely to shift; yet the cost of LPG is limiting the full transition to LPG in the near future (Gould and Urpelainen, 2018). Rice residues have traditionally been accepted as a suitable livestock fodder in Eastern India, unlike in Northwestern India (Erenstein and Thorpe, 2010), but feeding strategies are beginning to shift away from the use of rice residues as fodder (Bihar Livestock Master Plan, 2018). It is assumed that the changing herd size, herd type, and feeding practices will not only have implications for fodder demand, but will also have implications for emissions associated with enteric fermentation (Jha et al., 2011).

## 5 Conclusion

This study investigated the fate of rice crop residues and the influence of the field water environment on net GHG emissions in Eastern India using a combination of Tier 1, 2, and 3 modelling approaches. Results suggest that observed variations in field hydrology have an outsized effect on methane emissions, with wetter sites exhibiting higher net GHG emissions than drier sites across all residue management strategies, thus aligning with our hypothesis. Our hypothesis was correct given that in drier environments, biochar and incorporation pathways had the lowest net GHG fluxes. While in wetter environments, the biochar and burning pathways resulted in the lowest net GHG fluxes.

In relative and absolute terms, retaining residues through soil incorporation in comparatively wet sites results in very high emissions, suggesting that the current carbon ‘offset’ market's focus on increasing SOC stocks is inadequate for prioritizing and geographically targeting mitigation opportunities in Eastern India. Our results suggest that landscape-scale characterization of the hydrologic environment can be used to identify ‘safe’ places to add more carbon to soils while avoiding excessive CH_4_ emissions. Recommendations for no-burn alternatives need to be context-specific, considering what farmers can and cannot manage (i.e., water levels), with a clear expectation of outcomes across multiple sustainable development objectives (i.e., burning abatement and/or soil carbon sequestration).

## Funding

Funding for this study was provided by the Cornell Atkinson Center for Sustainability. This work was made possible, in part, by long-term funding from the Bill & Melinda Gates Foundation (BMGF grant numbers OPP1052535 and OPP1133205) to the Cereal Systems Initiative for South Asia (https://csisa.org/).

## CRediT authorship contribution statement

**Emily Urban Cordeiro:** Conceptualization, Methodology, Investigation, Formal analysis, Writing – original draft, preparation, Writing – review & editing, Supervision, Funding acquisition. **Laura Arenas-Calle:** Conceptualization, Methodology, Investigation, Formal analysis, Writing – review & editing. **Dominic Woolf:** Conceptualization, Methodology, Writing – review & editing, Supervision. **Sonam Sherpa:** Conceptualization, Investigation, Writing – review & editing, Supervision. **Shishpal Poonia:** Conceptualization, Investigation, Writing – review & editing. **Kritee Kritee:** Investigation, Writing – review & editing, Supervision. **Rachana Dubey:** Conceptualization, Writing – review & editing. **Amresh Choudhary:** Investigation, Writing – review & editing, Supervision. **Virender Kumar:** Conceptualization, Writing – review & editing. **Andrew McDonald:** Conceptualization, Methodology, Formal analysis, Writing – original draft, preparation, Writing – review & editing, Funding acquisition.

## Declaration of competing interest

The authors declare that they have no known competing financial interests or personal relationships that could have appeared to influence the work reported in this paper.

## Data Availability

Data will be made available on request.
