# Supplementary material for: The fate of rice crop residues and context-dependent greenhouse gas emissions: Model-based insights from Eastern India
Source: J Clean Prod. 2024 Jan 5;435:140240. doi: 10.1016/j.jclepro.2023.140240 (PMC10804972; doi:10.1016/j.jclepro.2023.140240)
Supplement: Multimedia component 1 [file mmc1.pdf]

Supplementary Information for

The fate of rice crop residues and context-dependent greenhouse gas emissions: model-based insights from Eastern India

Table of Contents: Supplementary Information

Figure S1	Field water pipe diagram and photographs
Figure S2	Cumulative distribution function (CDF) plot of daily precipitation for the three study years
Text S1	Methods: DNDC Monte Carlo
Table S1	Input for Monte Carlo simulations in DNDC
Table S2	Results of Monte Carlo simulations in DNDC
Text S2	Methods: DNDC sensitivity analysis
Table S3	Results of DNDC sensitivity analysis
Table S4	"Other" sources within the LCA contributions plot (Fig. 5 in manuscript)

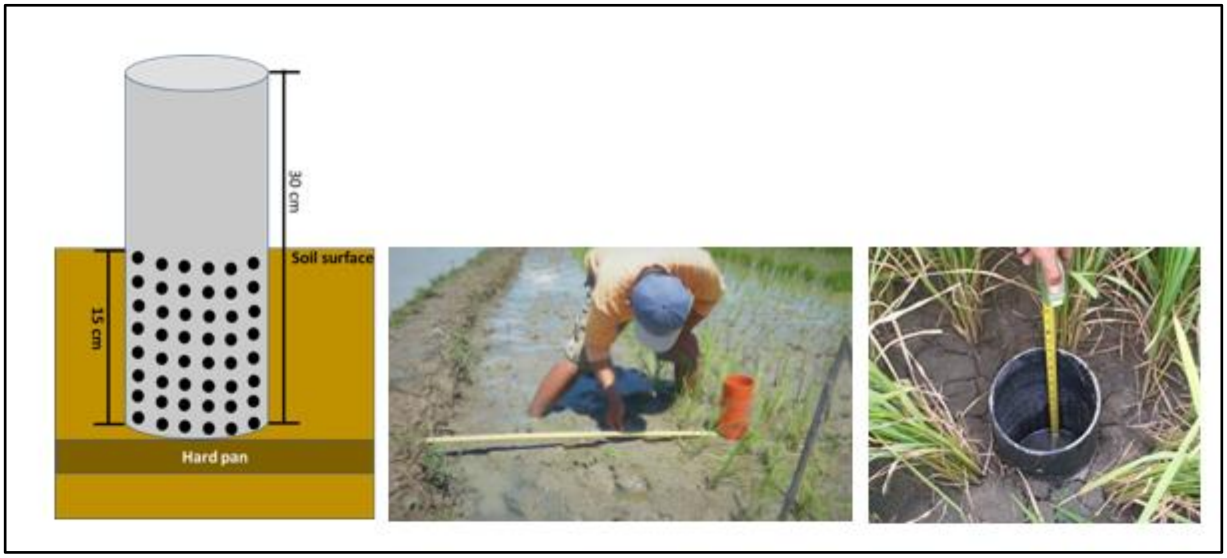

Figure S1. Field water pipe diagram and photographs

(left) Diagram depicting field pipe placement relative to the soil surface; (center) Photo demonstrating the distance between the paddy bund and a pipe; (right) Photo demonstrating how field water pipe measurements were taken.

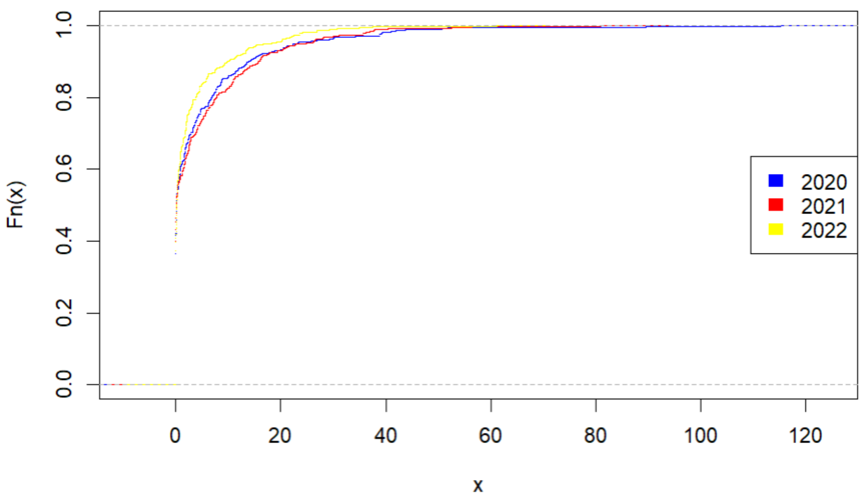

Figure S2. Cumulative distribution function (CDF) plot of daily precipitation for the three study years

The x-axis represents the daily precipitation values (mm) and y-axis represents the cumulative probability. Farmers and field staff anecdotally described 2020 as an 'average rainy season'.

Text S1. Methods: DNDC Monte Carlo

In this study, field hydrology is the factor with the largest uncertainty. This factor was extensively addressed through the comparison of three hydrological categories (i.e., dry, median, wet). The next area of uncertainty is the soils component (i.e., clay fraction, initial SOC, etc.) which was represented in the DNDC simulations.

There are several factors in the DNDC simulations that were treated as fixed soil, crop, and management attributes with a single value rather than ranges. To understand the uncertainty of these inputs to our overall results, Monte Carlo simulations were conducted with 32,768 replications (as specified by DNDC) for each of the three hydrology categories (i.e., dry, median, wet). These simulations were limited to five input parameters, which were selected based on the use of the DNDC model in the literature, to determine the uncertainty of the three response variables for this study (i.e., CH_4 flux, N_2O flux, and annual SOC stock change). The selected input parameters included clay fraction, initial SOC content (g C kg^{-1}), plant C:N ratio, fertilizer N rate (kg N ha^{-1}), and maximum yield fraction with the range specified by DNDC (Table S1). It is important to note that the results in the main paper are presented as annualized values over 100 years. The Monte Carlo exercise was conducted for 1 year.

Table S1. Input for Monte Carlo simulations in DNDC

**Input parameter**	**Baseline**	**Range***
Clay fraction	0.42	10%
Initial SOC content (g C kg^{-1})	5.2	10%
Plant C:N ratio	45 (rice); 40 (wheat)	20%
Fertilizer N rate (kg N ha^{-1})	169.41 (rice); 85.5 (wheat)	15%
Maximum yield fraction	0.46 (rice); 0.48 (wheat)	15%

*Monte Carlo parameterization is based on the fraction of the original input data (e.g., baseline)

Table S2. Results of Monte Carlo simulations in DNDC

Hydrology categories	CH_4 fluxes (Mg ha^{-1})		dSOC (Mg ha^{-1})		N_2O fluxes (Mg ha^{-1})	
	**Mean**	**SD**	**Mean**	**SD**	**Mean**	**SD**
Dry	0.03	<0.01	0.37	0.09	2.9e-5	5.7e-6
Median	0.08	0.01	0.34	0.09	2.9e-5	5.6e-6
Wet	0.11	0.02	0.33	0.09	2.8e-5	6.7e-6

Text S2. Methods: DNDC sensitivity analysis

A one-at-a-time sensitivity analysis was performed (*n*= 10 intervals) with the range of plausible values for our study area. The ranges were taken from field observations (*n*= 3,000) in Eastern India. Similar to the main simulations, the sensitivity simulations were run for 103 years, with the first three years removed in analysis for all three hydrologic conditions (i.e., dry, median, wet). From the literature, common DNDC parameters evaluated typically include initial SOC, bulk density, pH, soil texture (% clay fraction), and fertilizer application rates (Babu, Li, Frolking, Nayak, & Adhya, 2006; Qin, Zhao, Shi, Xu, & Yu, 2016; Tripathi et al., 2021). For interannual SOC change (dSOC), the five most influential parameters have shown to be initial SOC, bulk density, manure application, crop residue incorporated, and chemical fertilizer applied (Qin et al., 2016). However, for this sensitivity analysis, a limited number of input parameters were quantified in this exercise given the extensive computational and processing time required for the long-term time scale and three different hydrology categories. As such, three key parameters were selected, including fraction of clay, fraction of residue return, and initial SOC level.

The relative sensitivity index (SI) was calculated using Equation 1 for each response variable (Walker, Mitchell, Hirschi, & Johnsen, 2000).

$$SI = ((O_2 - O_1)/O_{avg})/((I_2 - I_1)/I_{avg}) \quad (1)$$

Where, for a given parameter,

O_1 = minimum model output value
O_2 = maximum model output value
O_{avg} = average value of O_1 and O_2
I_1 = minimum model input value
I_2 = maximum model input value
I_{avg} = average value of I_1 and I_2

Table S3. Results of the DNDC sensitivity analysis

**Tested input parameters**		**SI***					
		**CH_4 fluxes**			**SOC Stock**		
**Parameter**	**Range of values**	**Dry**	**Median**	**Wet**	**Dry**	**Median**	**Wet**
Clay fraction	0.02- 0.65	0.31	0.53	0.55	1.21	1.57	1.72
Initial SOC content (g C kg^{-1})	1.2- 9.5	0.13	0.08	0.07	2.14	2.42	2.83
Residue return fraction (%)	0- 100	0.25	0.39	0.40	0.85	0.87	0.90

**Relative sensitivity index (SI) is understood as the higher the absolute value is, the greater the influence on the response variable, either in a positive or negative direction*

Table S4. "Other" sources within the LCA contributions plot (Fig. 5 in manuscript)

**Pathway**	**Source**	**GHG Fluxes (Mg CO_2-e)**
Incorporation	N2O: Soil	0
Burning	N2O: Soil	0.01
Burning	CH4: Burn	0.1
Burning	N2O: Burn	0.57
Livestock	N2O: Soil	0
Livestock	CH4: Cookstove	0
Livestock	N2O: Cookstove	0
Livestock	CH4: Manure	0.24
Livestock	N2O: Manure	0.09
Biochar	N2O: Soil	0.01

References

Babu, Y. J., Li, C., Frolking, S., Nayak, D. R., & Adhya, T. K. (2006). Field validation of DNDC model for methane and nitrous oxide emissions from rice-based production systems of India. *Nutrient Cycling in Agroecosystems*, *74*(2), 157–174. https://doi.org/10.1007/s10705-005-6111-5

Qin, F., Zhao, Y., Shi, X., Xu, S., & Yu, D. (2016). Sensitivity and uncertainty analysis for the DeNitrification-DeComposition model, a case study of modeling soil organic carbon dynamics at a long-term observation site with a rice-bean rotation. *Computers and Electronics in Agriculture*, *124*, 263–272. https://doi.org/10.1016/j.compag.2016.04.017

Tripathi, R., Majhi, M., Sahu, S. G., Mohanty, S., Moharana, K. C., Shahid, M., … Nayak, A. K. (2021). Modelling the Spatial Variation of Methane and Nitrous Oxide Emission from Rice Fields Using DNDC Model. *Journal of the Indian Society of Remote Sensing*, *49*(12), 2919–2931. https://doi.org/10.1007/s12524-021-01433-3

Walker, S. E., Mitchell, J. K., Hirschi, M. C., & Johnsen, K. E. (2000). *SENSITIVITY ANALYSIS OF THE ROOT ZONE WATER QUALITY MODELS*. *43*(4), 841–846.
